# Do You See What I Mean? Corticospinal Excitability During Observation of Culture-Specific Gestures

**DOI:** 10.1371/journal.pone.0000626

**Published:** 2007-07-18

**Authors:** Istvan Molnar-Szakacs, Allan D. Wu, Francisco J. Robles, Marco Iacoboni

**Affiliations:** 1 Center for the Biology of Creativity, Semel Institute for Neuroscience and Human Behavior, University of California at Los Angeles, Los Angeles, California, United States of America; 2 Ahmanson-Lovelace Brain Mapping Center, University of California at Los Angeles, Los Angeles, California, United States of America; 3 FPR-UCLA Center for Culture, Brain and Development, University of California at Los Angeles, Los Angeles, California, United States of America; 4 Department of Psychiatry and Biobehavioral Sciences, University of California at Los Angeles, Los Angeles, California, United States of America; 5 Department of Neurology, University of California at Los Angeles, Los Angeles, California, United States of America; 6 Brain Research Institute, David Geffen School of Medicine, University of California at Los Angeles, Los Angeles, California, United States of America; Harvard Medical School, United States of America

## Abstract

People all over the world use their hands to communicate expressively. Autonomous gestures, also known as emblems, are highly social in nature, and convey conventionalized meaning without accompanying speech. To study the neural bases of cross-cultural social communication, we used single pulse transcranial magnetic stimulation (TMS) to measure corticospinal excitability (CSE) during observation of culture-specific emblems. Foreign Nicaraguan and familiar American emblems as well as meaningless control gestures were performed by both a Euro-American and a Nicaraguan actor. Euro-American participants demonstrated higher CSE during observation of the American compared to the Nicaraguan actor. This motor resonance phenomenon may reflect ethnic and cultural ingroup familiarity effects. However, participants also demonstrated a nearly significant (p = 0.053) actor by emblem interaction whereby both Nicaraguan and American emblems performed by the American actor elicited similar CSE, whereas Nicaraguan emblems performed by the Nicaraguan actor yielded higher CSE than American emblems. The latter result cannot be interpreted simply as an effect of ethnic ingroup familiarity. Thus, a likely explanation of these findings is that motor resonance is modulated by interacting biological and cultural factors.

## Introduction

Several different types of hand actions accompanying speech may be observed during social interactions enriching the communicative repertoire of a particular cultural community. These gestures belong to two broad categories: those accompanying speech or autonomous gestures [1], [2]. Autonomous gestures also known as emblems are highly social in nature, and convey conventionalized meaning without accompanying speech [3], [4]. Emblems have the property of being intentionally communicative, where the interlocutors both must be aware of the gesture to comprehend the message. Thus, the sender is fully aware of the meaning of the gesture they produce, while the perceiver can assume that the action was performed intentionally to convey information [1], [5]. The form of these gestures is arbitrary and their names are learned according to socially relevant and culturally specific codes [6]. Emblems can either accompany verbal material, or be used autonomously, and, in fact, they are often used to replace words in conversation. For example, we frequently use the thumbs-up gesture to indicate that something is ‘good’ in response to a verbal inquiry. Emblems are used particularly when environmental circumstances (loud noise) or voluntary choice (discretion) limit the use of the verbal channel. Thus, emblems maintain their semantic significance, even when presented in decontextualized ways, such as in photographs or videos [7].

The meaning of emblems is highly specific to particular linguistic groups, regions or cultures and their forms are replicated in the same form from person to person in a given cultural area [6]. Even during development, patterns of learned nonverbal behavior will reflect these differences. According to Birdwhistell (1970), the socially adaptive nature of human infants drives them to assume the conventions of the prevailing communication system of their environment [8]. In fact, Iverson and Goldin-Meadow (2005) have shown that gesture production facilitates language learning in infants and influences development of cognitive skills in general [9]–[11].

Although there is an increasing amount of research on how the human brain perceives and understands actions in general, at this stage we still know very little about how special classes of actions such as communicative hand gestures are understood. In particular, it remains an open question how cultural experience modulates the neural mechanisms of action perception and social communication. It has been proposed that action perception involves an internal simulation or replication of the observed action [12]. Research in monkeys has described a specific brain mechanism underlying this process. Mirror neurons are found in the premotor and parietal cortex of the macaque brain, and fire both when the monkey performs an action and when it observes another individual perform a similar action [13]–[20]. The ventral premotor cortex, and the inferior parietal lobule in the monkey form a fronto-parietal mirror neuron system critical to action understanding [20].

Neuroimaging methods are starting to give us a better understanding of the neural mechanisms of action perception in humans. Accumulating evidence has shown that perceiving other people's behaviors activates motoric representations in the brain similar to patterns of activity that are produced if we perform the same action ourselves [20]–[22]. Applying transcranial magnetic stimulation (TMS) to the motor cortex has revealed systematic changes in corticospinal excitability (CSE) while subjects watched meaningless finger movements [23]–[25], object oriented actions [26] and a hand performing pantomimes and meaningful hand signs [27]. These studies all indicate an involvement of the motor system of the observer even during passive perception of actions. Functional magnetic resonance imaging (fMRI) has been used to localize the neural network recruited during action perception. The observation of simple finger movements [28], [29], object directed actions [30], [31], pantomimes [32], [33] and meaningful hand signs [34], [35] appears to recruit a fronto-parietal network involving the posterior inferior frontal gyrus and adjacent ventral premotor cortex, as well as the inferior parietal lobule. Thus, it appears that in humans, as in monkeys, there exists a fronto-parietal mirror neuron network involved in the perception and representation of observed actions [20]. It has recently been proposed that this fronto-parietal mirror neuron system may also be involved in the perception of high-level, socially relevant communications such as intention understanding [30], music perception [36] and empathy [37].

As the cultural milieu determines which emblems become part of the gestural, communicative and social repertoire of an individual, this same environment exerts modulatory effects on the neural system for action understanding and social communication. In other words, cultural learning determines an individual's motor repertoire, and if the motor repertoire of two individuals is shared, there is a strong motor resonance between these individuals. In our case, a particular gesture may be part of the motor repertoire of a Nicaraguan, but not of a Euro-American individual. Presumably, if communicating individuals share a motor repertoire, at the neural level the same representations are activated in actor and observer, allowing them to interpret each other's actions and the communicative intent behind those actions. Conversely, there should be less internal simulation of an observed action, if that action is not part of the observer's motor repertoire.

**Figure 1:**
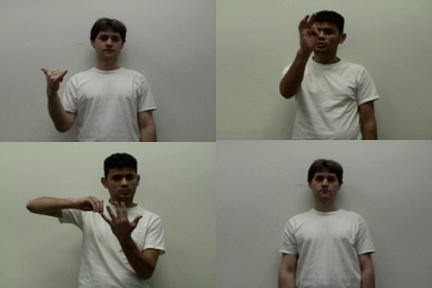
Examples of experimental stimuli. A) Euro-American actor performs the classic American ‘hang loose’ gesture. B) Nicaraguan actor performs a typical Nicaraguan gesture ‘I swear (promise)’ and C) one of the control gestures modified from the ASL sign for ‘berries’. D) Euro-American actor in the ‘static’ condition.

Motor resonance has been investigated in several neuroimaging studies. For example, watching biologically impossible actions seems to activate premotor areas less than possible actions [38] and similarly, watching an artificial hand in action evoked much less premotor activity than watching real hand actions [39], [40]. In a study of actions performed by conspecifics and non-conspecifics, Buccino and colleagues showed that actions belonging to the motor repertoire of the observer were more successful in eliciting activity within the fronto-parietal circuit for action representation than foreign actions [41]. In this study, we wanted to investigate the imprint of culture on the neural system for action representation and understanding. We used single pulse TMS to measure CSE in Euro-American participants while they watched a Euro-American or Nicaraguan actor perform both culturally familiar and foreign emblems (Figure 1).

Based on the above evidence of the influence of the observer's own motor repertoire on action perception, we hypothesized that a shared motor repertoire leads to more effective communication. Thus, we predicted that our Euro-American participants would show greatest facilitation of CSE during observation of the Euro-American actor. Properties of a unified perception/action system also predict that just as one's culturally acquired motor repertoire influences how one perceives actions, it also affects the performance of actions. Thus, a Nicaraguan performer's Nicaraguan gestures come from his culturally determined motor repertoire, but the same gestures are not part of the American performer's motor repertoire. We predicted an interaction of performer and gesture, reflecting the execution of actions from a familiar versus unfamiliar motor repertoire.

## Results

**Figure 2:**
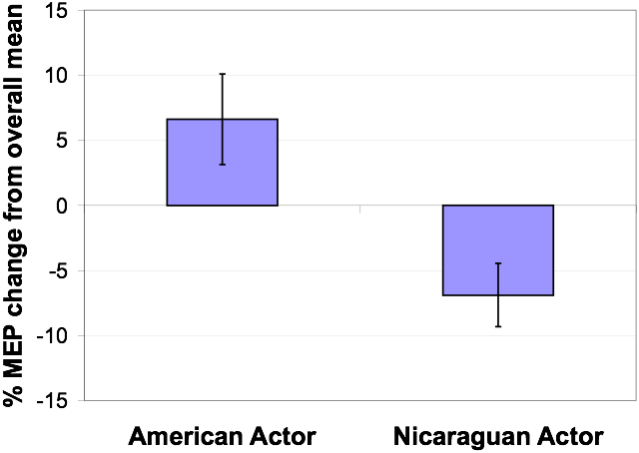
Main effect of performer (F(1,7) = 6.85, p<0.05). Percent change relative to the overall mean in motor evoked potential (MEP) responses recorded during observation of actions executed by the Euro-American actor versus Nicaraguan actor.

Changes in CSE were evaluated using a repeated-measures ANOVA, with actor (Euro-American, Nicaraguan), stimulus type (American emblems, Nicaraguan emblems, control ASL, static) and hemisphere (Left hemisphere (LH), right hemisphere (RH)) as within subject factors. We found a significant main effect of actor (F(1,7) = 6.85, p<0.05), due to higher CSE for observing the Euro-American actor compared to the Nicaraguan actor (Figure 2).

ANOVA also revealed a nearly significant performer by gesture interaction (F(3,5) = 5.24, p = 0.053), Figure 3. Post-hoc paired t-tests show no differences in CSE while observing emblems performed by the Euro-American actor. In contrast, the observation of Nicaraguan emblems yielded higher CSE than American emblems (p = 0.004) and control ASL signs (p = 0.028) when performed by the Nicaraguan actor. We found no further significant main effects or interactions.

**Figure:**
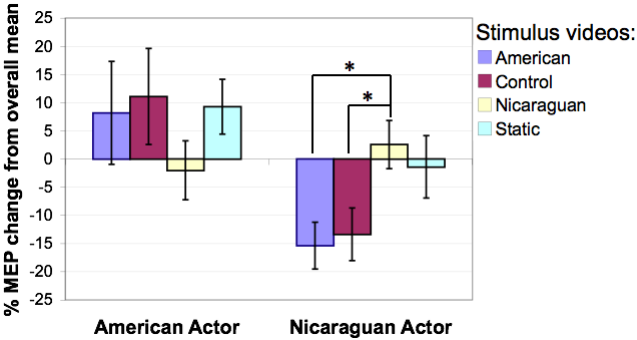
Gesture type x Performer interaction (F(3,5) = 5.24, p = 0.053). Post-hoc paired t-tests on percent change relative to the overall mean in motor evoked potential (MEP) responses show no differences in CSE while observing emblems performed by the Euro-American actor. In contrast, the observation of Nicaraguan emblems yielded higher CSE than American emblems (P = 0.004) and control ASL signs (P = 0.028) when performed by the Nicaraguan actor.

## Discussion

In this study, we used culture-specific, meaningful non-verbal hand gestures to investigate whether motor resonance during action observation is modulated by cultural factors. Indeed, the observation of actions performed by an individual of one's cultural and ethnic ingroup increases CSE, compared to observing actions performed by an outgroup member. While this modulation of CSE may be attributed to ethnic ingroup familiarity, the interaction between actor and emblem type cannot be accounted for by such familiarity. We propose that a plausible explanation of these findings is that unconscious motor resonance mechanisms are modulated by interacting biological and cultural factors.

While observing the actions of an ethnic and cultural ingroup member, we show stronger motor resonance. This novel result is interesting because it implicates one's own motor system in the perception of ingroup versus outgroup members, independent of observed motor actions. Our data showing increased CSE at the implicit, individual level, are in line with previously described effects at a more explicit and social level. Indeed, differential perception of ingroup versus outgroup members has been described extensively in the literature (for recent reviews see: [42]–[44]). Persons tend to have higher empathy for ingroup members [45] and favor them in reward allocations [46] and in esteem[47]. Cognitively, people remember more detailed information about ingroup members than outgroup members [48]. This bias leads people to encode the observed behaviors of ingroup and outgroup members at different levels of abstraction [49]. For example, undesirable actions of outgroup members are presumed to be of intentional and dispositional origin (‘she is hostile’), compared to identical behaviors of ingroup members (‘she slapped the girl’). The converse is true for desirable actions, which are encoded at more concrete levels for outgroup members (‘she walked the old man across the street’) relative to the same behaviors in ingroup members (‘she is kind’) [50]. Thus, it appears that neural systems supporting memory, empathy and general cognitions encode information related to ingroup versus outgroup members differently. One novel contribution of the current study is our finding that the human mirror neuron system specifically, is differentially sensitive to ingroup versus outgroup members.

This finding is particularly interesting in light of recent data implicating the fronto-parietal human mirror neuron system in self-other distinction [51], [52]. Based on recent findings, it has been proposed that a mechanism similar to that which enables the understanding of the actions of others also allows identification of other agents by mapping their physical characteristics onto one's own motor repertoire [53]. Our data agree with this proposal, and provide additional evidence that a motor resonance mechanism mediates intersubjective communication and social communication in general.

In nature, as in our study, biological factors such as ethnic ingroup membership and cultural factors such as motor repertoire are inextricably linked, especially in investigations of highly culture-specific actions such as emblems [6]. While this makes interpretation of data somewhat more complex, it does more accurately reflect what our brains process in the real world. Our current results show that ethnic ingroup membership *and* a culturally learned motor repertoire influence the brain's responses to observed actions, specifically actions used in social communication. In functional terms at the neural level, the mirror neuron system is involved in predicting action goals [20] and providing an ongoing simulation of the motoric complexity of observed actions [31] while maintaining a representation of the intention behind those actions [30]. The present data show that while this system for action representation is responding to observed actions, the response is modulated not only by the kind of action that is observed, but also by who is performing that action.

Our initial hypothesis, based on the neuroimaging literature on action perception, predicted that a shared motor repertoire leads to more effective communication. Thus, we predicted that our Euro-American participants would show facilitation of CSE during observation of the Euro-American actor due to a shared motor repertoire. This prediction was in fact borne out, as shown by our main effect of actor, however, the neural processes giving rise to this effect may not simply be due to the perception of familiar actions. Our results are more nuanced showing that the human mirror neuron system may identify elements in a shared motor repertoire, but it is also sensitive to ethnic group membership. This is evidenced in the performer by gesture interaction, showing that even if familiar actions are observed, it does not translate into stronger motor resonance, as indexed by an increase in CSE.

Additional support for this interaction of ethnicity and one's motor repertoire is the finding that the American emblems performed by the Nicaraguan actor did not lead to facilitation of CSE, but rather to a decrease. The decrease in CSE during observation of a Nicaraguan actor performing American emblems is likely due to a perceived incongruence between the actor and the action they are performing. Our American participants observing an ethnic outgroup member perform actions that the participants themselves know well, may trigger a ‘differentiation’ response rather than one of ‘identification’ with the actor. Such a response is likely due to an interaction of biological factors (ethnicity) and cultural factors (learned motor repertoire). Considering this finding another way, it is interesting to note that Nicaraguan emblems performed by the Nicaraguan actor did not lead to a decrease in CSE, and may indicate that the socially relevant nature of these gestures were evident to our participants (even without semantic comprehension), such that they may have tried to map these gestures onto their own motor repertoire.

This modulation of CSE while observing the Nicaraguan actor performing his own culturally learned emblems is intriguing, and suggests modulation of motor resonance mechanisms. This finding is similar to our recent data showing stronger recruitment of fronto-parietal mirror neuron regions during observation of complex hierarchical action sequences of increasing motoric complexity and increased reaction times during construction of such complex sequences [31]. It suggests that motor resonance, while an implicit parameter of action recognition, is a nuanced one, conveying subtle learned differences in motor fluency.

Due to the close relationship of gesture and language[54] and the traditional view of the left hemisphere being language-dominant[55] it is important for us to consider the issue of laterality. In this study, we did not find any main effect or interaction with hemisphere. Consistent with our results, previous work examining the lateralization of the human mirror neuron system during hand action observation using TMS[24] and fMRI[56] has found that the system for action representation is on the whole bilateral. This was also the finding of the reanalysis of a large dataset of functional imaging studies (58 subjects) involving observation and imitation of simple finger movements[29]. A recent study of a split-brain patient assessed laterality of the mirror neuron system using TMS, and found that while the left hemisphere of the patient showed increased CSE during action observation, the right hemisphere did not[57]. However, a control group of normal subjects showed parallel increases in CSE in both hemispheres, indicating that in fact, action representation recruits both hemispheres.

We hope that this work will stimulate further experiments to investigate the effect of cultural learning on the motor system using participants from two different cultures. In fact, we also tried to recruit Nicaraguan participants for the present study from the Los Angeles area. However, due to the large variability in exposure to American culture, as well as varying degrees of assimilation and acculturation, it became evident that we would be unable to enroll participants that were equally naïve with respect to American gestures as the American participants were with the Nicaraguan gestures. This issue highlights the increasingly more relevant effects of globalization on research. A future experiment with participants from two cultures should help disentangle the effects of biological factors (ethnic ingroup membership) and cultural factors (motor repertoire) on the perception of action. A caveat with the current study is the limited number of participants, thus conclusions must be drawn carefully; however, several other TMS studies have also used eight or fewer participants to study cognitive phenomena [52], [58]–[61].

In conclusion, our findings suggest that the neural substrates of action recognition and social communication may be tuned to both ethnic identity and cultural experience. We have shown that observing the actions of an individual who is an ethnic ingroup member and shares a culturally acquired motor repertoire yields higher motor resonance, compared to observing an individual who is an ethnic outgroup member and has an unfamiliar culturally acquired motor repertoire. Our findings suggest that the human mirror neuron system is implicated in distinguishing ingroup versus outgroup members, and this same neural mechanism is involved in representing culturally learned actions. These findings may have broad implications for motor skill and language learning, intergroup communication, as well as the study of intergroup attitudes and stereotyping.

## Methods

### Participants

Eight Euro-American participants (4 males) were recruited for this study approved by the UCLA Institutional Review Board, conforming to The Code of Ethics of the World Medical Association (Declaration of Helsinki). Written informed consent was obtained from all participants. All participants were right-handed according to a modified Edinburgh Handedness Questionnaire [62]. The participants were screened for neurological, psychiatric and medical problems, drug use, as well as contraindications to TMS [63]. Participants had a mean age of 20.5 years (range 18–24 years), and were all native English speakers.

### Stimuli

As stimuli, we showed 5 second long digital video clips of American and Nicaraguan emblems. As a control condition for familiarity and emblem type, we used modified signs from American Sign Language (ASL). In a fourth condition, participants observed video clips of the actors standing still. The American emblems included the: ‘thumbtwiddle’, ‘shamefingers’, ‘hang loose’ and ‘OK’, Figure 1a. The Nicaraguan emblems included: ‘play marbles’, ‘get caught’, ‘I swear’ and ‘neat/well done’, Figure 1b. Emblems maintain their referential power even when presented without elements of the relevant semantic context [7], thus participants should have no problem understanding our stimuli from the video clips. To ensure this, we pre-tested the meaningfulness of our stimulus set on an independent group of participants. Ten out of 10 Euro-Americans (tested in Los Angeles) recognized and labeled all four American emblems correctly and knew none of the Nicaraguan emblems. Six out of 6 Nicaraguan participants (tested in Nicaragua) recognized and labeled all four Nicaraguan emblems correctly and did not identify any of the American emblems correctly. None of the 16 participants questioned in the independent group recognized ASL signs, and had no previous experience with sign language. The ASL signs we modified included: ‘chain’, ‘pick berries’, ‘buy’ and ‘advice’, Figure 1c. Each stimulus type was performed by both an Euro-American and a Nicaraguan actor. We tried to match external characteristics of the actors such as gender, age, height and build, and they were both dressed in white T-shirts, filmed against a plain white background. We recorded only the upper part of the body, allowing for adequate gesture space around the body to perform the stimuli. To prevent interpretation of information from facial expression, the actors were asked to keep their facial expressions neutral. While external characteristics were matched as much as possible, phenotypic signs of ethnicity were present (darker skin color of the Nicaraguan actor). Furthermore, the Nicaraguan actor was a first generation immigrant to the US who spent all his childhood and adolescence in Nicaragua with virtually no American influence due to government imposed censorship on US media and television.

### TMS

Participants were seated in front of a computer monitor, with their head in a chin rest and fitted with a neck brace to minimize head movement. Single-pulse transcranial magnetic stimulation was delivered through a 9×5 cm corticoil using a High Speed MES-10 stimulator (Cadwell Laboratories, Inc.) over the right or left primary motor cortex. Motor-evoked potentials (MEPs) were recorded from the first dorsal interosseous (FDI) muscle of each hand. The coil was held tangentially on the scalp, approximately perpendicular to the central sulcus, 45^0^ from the anterior-posterior axis, with the handle pointing posteriorly over the optimal spot for eliciting MEPs in the contralateral FDI muscle [64] (amplification x2000-5000, band-pass filter 0.3–1000 Hz, digitization sampling rate of 2 kHz/channel). MEPs were recorded using 10-mm Ag/AgCl surface electrodes with the active electrode placed over the motor point and the reference electrode placed over the tendon of the muscle.

The resting motor threshold (MT) was assessed according to conventional criteria, i.e. the minimal stimulator output that induced MEPs of at least 50-µV in five out of ten trials [65], and determined separately for each hemisphere. Output of the stimulator was subsequently adjusted to 10% above resting motor threshold to produce an MEP with peak-to-peak amplitude of at least 50-µV during the experimental conditions. Background EMG activity was monitored to ensure that participants maintained relaxed hand muscles during all tasks.

To assess hemispheric differences in the change of the size of the MEP during the experimental tasks, each participant was stimulated over the left and right hemisphere. The order of stimulation sites was counterbalanced between participants. For each hemisphere, 64 trials were recorded in two runs of 32 trials: 4 American videos, 4 Nicaraguan videos, 4 control videos and 4 static videos performed by the Nicaraguan actor and the same stimuli performed by the Euro-American actor. Each of the 5 second long video clips were centrally presented, in color on a 21″ Optiquest V115 computer monitor, and the TMS pulse was delivered 4 seconds after stimulus onset. At the end of a clip, the video was replaced by a green square, prompting participants to give a verbal response. Participants were asked to watch the presented actions and after each trial, at the appearance of the green square, to quietly say ‘yes’ if they knew the meaning of the presented emblem, or ‘no’ if the emblem was unfamiliar, or during static videos. Each trial was followed by 5 seconds of rest. The order of stimuli was fully randomized within each run of each participant.

All data were analyzed off-line with a MATLAB (Mathworks, MA) software tool for analysis of time-series data (dataWizard)[66]. Raw MEP amplitudes were recorded as maximal peak-to-peak amplitudes following TMS. MEPs with amplitudes ±2 standard deviations away from the mean value of each participant's hemispheric mean were discarded. MEP amplitudes were then normalized to the overall mean MEP obtained for that participant, in each hemisphere across all conditions. We did this to account for intra-subject variability in motor thresholds of the two hemispheres and inter-subject variability in the size of the MEP. Peak-to-peak amplitudes of the MEPs were averaged and mean amplitudes obtained for each experimental condition in each hemisphere of individual participants.
